# Circulating Matrix Metalloproteinase-9 Is Associated with Cardiovascular Risk Factors in a Middle-Aged Normal Population

**DOI:** 10.1371/journal.pone.0001774

**Published:** 2008-03-12

**Authors:** Peter Garvin, Lennart Nilsson, John Carstensen, Lena Jonasson, Margareta Kristenson

**Affiliations:** 1 Division of Community Medicine, Department of Medical and Health Sciences, Linköping University, Linköping, Sweden; 2 Division of Cardiology, Department of Medical and Health Sciences, Linköping University, Linköping, Sweden; 3 Division of Health and Society, Department of Medical and Health Sciences, Linköping University, Linköping, Sweden; University of Sheffield, United Kingdom

## Abstract

**Background:**

Elevated levels of circulating matrix metalloproteinase-9 (MMP-9) have been demonstrated in patients with established coronary artery disease (CAD). The aim of this study was to analyse levels of MMP-9 in a population free from symptomatic CAD and investigate their associations with cardiovascular (CV) risk factors, including C-reactive protein (CRP).

**Methods:**

A cross-sectional study was performed in a population based random sample aged 45–69 (n = 345, 50% women). MMP-9 levels were measured in EDTA-plasma using an ELISA-method. CV risk factors were measured using questionnaires and standard laboratory methods.

**Results:**

Plasma MMP-9 was detectable in all participants, mean 38.9 ng/mL (SD 22.1 ng/mL). Among individuals without reported symptomatic CAD a positive association (p<0.001) was seen, for both men and women, of MMP-9 levels regarding total risk load of eight CV risk factors i.e. blood pressure, dyslipidemia, diabetes, obesity, smoking, alcohol intake, physical activity and fruit and vegetable intake. The association was significant also after adjustment for CRP, and was not driven by a single risk factor alone. In regression models adjusted for age, sex, smoking, alcohol intake and CRP, elevated MMP-9 levels were independently positively associated with systolic blood pressure (p = 0.037), smoking (p<0.001), alcohol intake (p = 0.003) and CRP (p<0.001). The correlation coefficient between MMP-9 and CRP was r = 0.24 (p<0.001).

**Conclusions:**

In a population without reported symptomatic CAD, MMP-9 levels were associated with total CV risk load as well as with single risk factors. This was found also after adjustment for CRP.

## Introduction

It is today widely accepted that inflammatory processes are involved in plaque rupture preceding acute coronary events [1], [2]. In particular, pro-inflammatory cytokines and acute phase reactants including C-reactive protein (CRP), have been used as biomarkers and predictors for acute coronary syndromes (ACS) [3]–[6]. In parallel, pro-inflammatory cytokines such as interleukin-1 (IL-1) and tissue necrosis factor-α (TNF-α) have been shown to up-regulate matrix metalloproteinase-9 (MMP-9) [7], [8]. MMP-9 is considered to be a key determinant of extracellular matrix degradation, having collagen as the main substrate. It is to date the most studied enzyme in the MMP-family regarding cardiovascular diseases. Increased concentrations and activity of MMP-9 have been observed in human atherosclerotic vulnerable plaques with high inflammatory activity [9]–[12], suggesting a role in matrix degradation and plaque rupture.

So far, most studies exploring elevated levels of MMP-9 and its association to coronary artery disease (CAD) have been based on a experimental or clinical design, the latter showing elevated levels of plasma MMP-9 in patients with CAD [13]–[18]. While there is a number of experimental or cross-sectional studies in selected populations that associates elevated levels of MMP-9 to CAD risk factors before onset of disease e.g. smoking [19], alcohol [20], and hypertension [21], [22], little is still known about the distribution of MMP-9 in the general population. In a population-based study in the Framingham cohort, MMP-9 could be detected in plasma in only 20% of the participants, thus suggesting that MMP-9 was unlikely to be an informative biomarker in a low-risk population [23].

In the present study, we hypothesized that circulating levels of MMP-9 were independently related to cardiovascular risk factors, before onset of ACS. The aim with the study were divided into three steps: 1) To test the association of levels of MMP-9 and a total risk load of eight cardiovascular (CV) risk factors, i.e. blood pressure, dyslipidemia, diabetes, obesity, smoking, alcohol intake, physical activity and fruit and vegetable intake. 2) To test the association of levels of MMP-9 and each of the risk factors mentioned. 3) To evaluate the associations above after adjustment for CRP. In addition, analyses were adjusted for Tissue Inhibitor of Metallo Proteinase-1 (TIMP-1), one endogenous inhibitor of MMP-9.

## Methods

### Study population and design

Participants were recruited from the Life conditions, Stress and Health (LSH) study, a population based study, aiming at testing if psychobiological pathways mediate the association between socio-economic status and incident cardiovascular disease [24]. It was conducted in the county of Östergötland in the southeast of Sweden, with the study base defined as the population living in the catchment area for any of the ten collaborating Primary Health Care Centres (PHCC's). Participants were evenly distributed by sex and age, ranging from 45 to 69 years at enrolment. Exclusion criteria were self-reported severe disease that hindered the possibility to participate, e.g. terminal cancer, severe dementia and psychiatric disorders. Data collection was conducted in late 2003 and early 2004, constituted by a brief health examination, collection of blood samples and questionnaires. Participants with clinical symptoms of infection were instructed to come back to a PHCC after recovery.

The response rate for the initial baseline was 62.5%. The sample was representative for the population in terms of educational attainment, employment rates and immigrant status. The study design was approved by the ethical committee of the medical faculty, Linköping University, and written consent was obtained from all participants.

402 individuals, randomly chosen within the first 1 000 participants in the LSH-study were included in this sub-study, without any stratification criteria such as sex, age or other properties.

There was no potential conflict of interest for the authors or funders of this study.

### Data from the physical examinations

All nurses collecting data were trained together and the laboratory equipment used was calibrated to ensure standardization. The participants came to the PHCC's in the morning to minimize effects of diurnal variation, all in a fasting state. Heart rate, systolic and diastolic blood pressure (SBP and DBP) were measured in a sitting position in two minutes interval after five minutes of rest, using the mean of second and the third measurement (Omron M5-1 digital). Weight, height and waist-hip measures in standing position were collected. Plasma glucose (HemoCue), triglycerides and lipids (ADVIA 1650) were analysed directly after sample collection and LDL-cholesterol was calculated using Friedewalds formula [25]. For full information on data collection, see Hollman and Kristenson [24].

### Biochemical analyses

The concentrations of MMP-9 and tissue inhibitor of metalloproteinase 1 (TIMP-1) were measured in EDTA-plasma [26], by human Biotrak ELISA systems (Amersham Biosciences, Uppsala, Sweden). The assay for MMP-9 measures MMP-9, Pro-MMP-9 and the ProMMP-9/TIMP-1 complex. The lower detection limit was 0.6 ng/mL, interassay coefficient of variance (CV) was 7.2 to 7.9%. TIMP-1 was measured in half of the participants (n = 201), The assay recognises free TIMP-1 and TIMP-1 complexed with MMPs, with a lower detection limit of 1.25 ng/mL and a CV of 6.8%. C-reactive protein (CRP) was measured in serum by a highly sensitive latex-enhanced turbidimetric immunoassay (Roche Diagnostics GmbH, Vienna, Austria) with a lower detection limit of 0.03 mg/L and CV of 1.7%. Aliquotes of plasma and serum (0.5 mL) were stored in −70° Celsius approximately 18 months before laboratory analysis.

The analyses were performed in two steps, where preliminary statistical analyses were performed on half of the participants (n = 201), before deciding if analysis should be performed on the entire sub-sample.

### Data from questionnaires

The participants responded to a questionnaire including questions on smoking, alcohol intake, physical activity, fruit and vegetable intake, ongoing medication and self-reported medical diagnoses. To measure symptomatic CAD, the question “Have you ever had any event of myocardial infraction or angina pectoris diagnosed by a medical doctor? (Yes/No/Don't know)” was used. A similar construct was used to capture self-reported diabetes. Alcohol intake was based on a food frequency questionnaire adopted from the Swedish Mammography Cohort [27], combining frequency and typical amounts of different beverages. The intake of fruit and vegetables utilized the same food frequency questionnaire [27], wherefrom a sum score of gram fruit and vegetables per day was calculated. The participants were divided into three subgroups: low intake (less than mean minus one SD), moderate (mean plus/minus one SD) and high intake (more than mean plus one SD). The measurements of physical activity were adopted from the population surveys conducted by the Public Health Science Centre in the county of Östergötland. Two questions regarding daily activity were combined into an index with four categories: inactive, low, moderate and high level of physical activity [28]. Smoking and alcohol intake were quantified, whereas the subgroups stratified on fruit and vegetable intake and physical activity, respectively, were used as ordinal categories, as attempts to quantify to any further extent would be based on assumptions that cannot be validated in our data set.

### Statistical analysis

Circulating levels of MMP-9 was set as the main outcome measure. As previous CAD event might elevate levels of MMP-9 [13]–[18], and statins might lower levels of MMP-9 [29], [30], participants with a self-reported history of CAD and participants using hypolipidaemic drugs were excluded from the analyses. The analysis were principally divided into three steps: 1) In regression models, total risk load was tested, using dichotomies of the eight risk factors blood pressure, LDL/HDL-ratio, diabetes, obesity, smoking, alcohol intake, physical activity and fruit and vegetable intake. To ensure that the association was not driven by a single risk factor alone, risk load models excluding one risk factor at a time were tested as well. 2) Studying single risk factors, linear regression models adjusting for age and sex were performed on SD increment based on SD within the study population for each independent variable. Multivariate regression models adjusting for age, sex and variables with highly significant associations to MMP-9 in univariate analyses (p<0.001) were performed in addition. 3) Both the total risk load model and the regression models on single risk factors were performed with and without adjustment for CRP. Separate models for men and women were also performed for all associations studied. In a sub-sample (n = 172) additional adjustment for Tissue Inhibitor of Metallo Proteinase-1 (TIMP-1) were performed, to evaluate the effect of one endogenous inhibitor of MMP-9.

Aiming at minimizing bias of reversed causation, participants that reported they recently had changed their behavior due to health related problems were excluded when calculating means for behavioral risk factors. In regression models participants that recently had quit smoking or recently had reduced alcohol intake were categorized as top ordinal category. However, questionnaires where not specific enough to perform this action regarding physical exercise or fruit and vegetable intake.

A 2-sided probability value of p ≤0.05 was considered as statistically significant. ANOVA-analyses were used to outline any significant differences over ordinal categories, t-tests were used in dichotomies.

Analyses were performed in STATA statistical software, release 6.0, Stata Corporation, and SPSS for Windows statistical software, release 14.0.2, SPSS Inc.

## Results

### Clinical characteristics and plasma MMP-9 levels

MMP-9 could be detected in all participants (n = 402). In total, the mean for MMP-9 was 40.2 ng/mL with a range from 2.9 to 246.1 ng/mL (SD = 26.7), and no difference of mean level between the first (n = 201) and the second (n = 201) run of analyses (p = 0.491). There were significantly higher levels of MMP-9 in the subgroup with self-reported CAD (n = 16) compared to participants without reported symptomatic CAD (mean: 52.7 (SD 34.5) vs 38.7 (SD 22.1) ng/mL, p = 0.017). To allow for analysis on participants without symptomatic CAD, this sub-group (n = 16) was excluded in further analyses along with participants unsure of diagnosis and missing values on self reported CAD (n = 21). Further, the associations of MMP-9 with use of hypolipidaemic and antihypertensive agents, respectively, were tested in regressions adjusting for sex, age and CV risk factors. As there were a significant association with hypolipidaemic drugs (n = 20, beta in regression when adjusting for age, sex and risk factors: −11.9 ng/mL, p = 0.023), these participants were excluded. No significant association could be seen for antihypertensive drugs (p = 0.294), why participants on such medication were kept in the analyses. After all exclusions (previous self-reported CAD diagnosis and use of hypolipidaemic drugs), 345 participants remained. Their clinical and laboratory characteristics are shown in Table 1. The mean of MMP-9 in the remaining group was 38.9 ng/mL with a range from 2.9 to 143.9 ng/mL (SD 22.1). In univariate analysis, women (n = 175) had significantly lower mean of MMP-9 than men (n = 170) (34.7 ng/mL vs 43.2 ng/mL, p<0.001). No significant trend could be seen over age.

**Table 1:** Characteristics of study sample, n = 345.

		Men (n = 170)	Women (n = 175)
**Clinical and laboratory characteristics**	Age (yrs)	56.4±7.2	56.4±7.2
	BMI (kg/m^2^)	27.1±3.9	26.0±4.7
	waist circumference (cm)	97.6±10.7	84.3±11.7
	Diabetes (%)	7.7 (n = 13)	5.2 (n = 9)
	SBP (mm Hg)	135.0±18.2	128.2±20.0
	DBP (mm Hg)	85.1±11.7	82.0±11.3
	Antihypertensive treatment (%)	14.7 (n = 25)	13.7 (n = 24)
	Heart rate (bpm)	64.5±10.7	68.2±9.8
	Total cholesterol (mmol/L)	5.5±0.9	5.6±0.9
	LDL (mmol/L)	3.5±0.9	3.4±0.8
	HDL (mmol/L)	1.4±0.3	1.7±0.3
	LDL/HDL cholesterol ratio	2.5±0.7	2.1±0.6
	Triglycerides (mmol/L)	1.5±0.9	1.1±0.6
	CRP (mg/L)	0.8 (0.2–2.1)	0.7 (0.2–1.9)
	TIMP-1 (ng/mL)	288.2 (254.2–314.8)	268.9 (244.9–300.3)
**Behavioral characteristics**	Smoking (%)	17.9 (n = 30)	15.7 (n = 27)
	Drinking (%)	15.3 (n = 26)	2.9 (n = 5)
	High level of physical activity (%)	16.2 (n = 27)	19.0 (n = 33)
	High fruit intake (%)	8.3 (n = 14)	12.6 (n = 22)
**Outcome measures**	Detectable MMP-9 (%)	100	100
	Plasma MMP-9 (ng/mL)	43.2±24.2	34.7±19.1

Participants with reported symptomatic CAD and participants using statins are excluded. Analysis on TIMP-1 performed on a sub-sample, n = 172. All values are mean±SD or percentages, except CRP and TIMP-1, expressed as median and interquartile range.

### Relations between plasma MMP-9 levels and total load of risk factors

**Figure 1:**
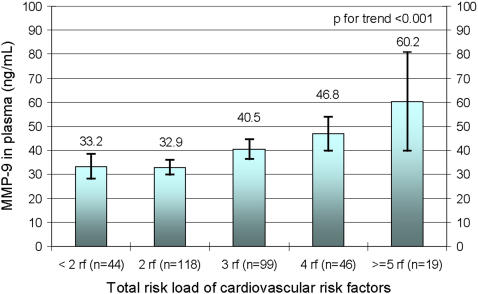
MMP-9 in relation to total risk load. Dichotomies of eight risk factors are taken into account in the analysis: n = 326 with full information on every variable. Blood pressure (SBP>160 and/or DBP>100 mm Hg and/or using antihypertensive agents, n = 81), LDL/HDL (ratio >3, n = 54), obesity (BMI>30, n = 67), diabetes (yes, n = 22), smoking (yes, n = 57), alcohol intake (>170 grams/week, n = 37), low physical activity (all other than top ordinal category, n = 272), low fruit and vegetables intake (all other than top ordinal category n = 307). Mean and 95% confidence intervals.

An analysis of MMP-9 levels over total risk load was performed, presented in Figure 1. There were 326 participants with information in all of the eight risk factors included. We found a highly significant association between plasma MMP-9 levels and number of risk factors added (+5.7 ng/mL (95% CI 3.6;7.7) per additional risk factor. p<0.001). The findings were persistent when divided by sex (not shown in figure, men n = 157 p = 0.005, women n = 167 p<0.001). After adjustment for CRP, the association between level of MMP-9 and number of risk factors were still significant (+4.7 ng/mL (95% CI 2.6;6.8) per additional risk factor. p<0.001), with persistent findings when divided by sex (men p = 0.004, women p = 0.001). Adjusting for TIMP-1 in a sub-analysis did not alter the results, as there neither was any significant association between TIMP-1 and total risk load (p = 0.576) nor between TIMP-1 and MMP-9 (p = 0.972).

Further, the association to the total risk load was not driven by a single risk factor. When removing one risk factor at a time from the total risk load, the associations between risk load and MMP-9 were highly significant (p<0.001) in all eight models tested, regardless of adjusting for CRP or not.

### Relations of levels of MMP-9 and single risk factors

**Figure 2:**
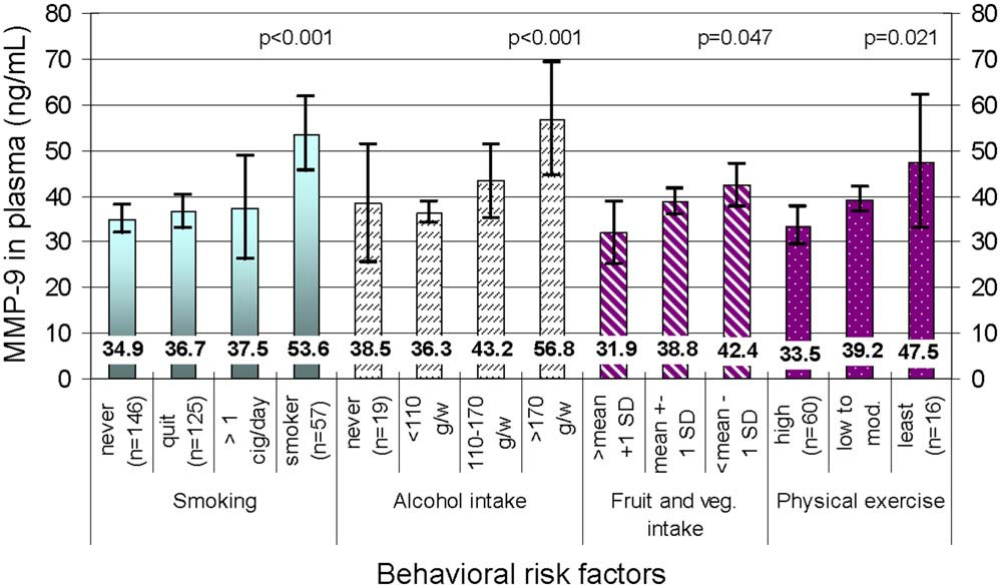
MMP-9 in relation to ordinal categories of smoking, alcohol intake, fruit and vegetable intake and physical exercise, respectively. Mean and 95% confidence intervals.

Distribution of MMP-9 over ordinal categories of the behavioral risk factors smoking, alcohol intake, fruit and vegetable intake and physical exercise are presented in figure 2. There were significant differences of MMP-9 levels between the ordinal categories of all four factors tested. The smoking group reported a mean of 12.7 cigarettes per day (SD 6.5 cigarettes per day). The reported mean alcohol intake in the ordinal categories were 34.g/week (SD 29 g/week), 138 g/week (SD 14 g/week) and 270 g/week (SD 313 g/week), respectively. Regression models are presented in table 2. Adjusting for age and sex, level of MMP-9 was significantly associated to the single risk factors waist circumference, SBP, heart rate, low HDL, triglycerides and CRP apart from each of the four behavioral factors tested. When adjusting for age, sex and CRP (data not shown), level of MMP-9 was significantly to SBP, triglycerides, each of the four behavioral factors tested and CRP. In multivariate analyses adjusting for age, sex, smoking, alcohol intake and CRP, level of MMP-9 was independently associated with SBP, smoking, alcohol intake and CRP, respectively.

**Table 2:** Linear regressions for MMP-9 as dependent variable.

	a) Age and sex		b) Age, sex, smoking and alcohol intake		c) Age, sex, smoking alcohol intake and CRP	
Variable (SD increment or category)	Beta (95% CI)	p-value	Beta (95% CI)	p-value	Beta (95% CI)	p-value
BMI, (4.3 kg/m^2^)	2.3 (−0.0;4.4)	0.053	**2.3 (0.1;4.7)**	**0.038**	0.4 (−2.0;2.8)	0.731
Waist circumference (13.0 cm)	**3.4 (0.7;6.1)**	**0.013**	**3.0 (0.4;5.7)**	**0.023**	0.7 (−2.1;3.5)	0.624
Diabetes (y/n)	−7.8 (−17.3;1.7)	0.104	−5.3 (−15.2;4.5)	0.282	−6.7 (−16.2;2.8)	0.165
SBP (19 mm Hg)	**2.9 (0.4;5.4)**	**0.022**	**3.0 (0.6;5.5)**	**0.014**	**2.5 (0.1;4.9)**	**0.037**
DBP (12 mm Hg)	2.1 (−0.2;4.5)	0.075	2.0 (−0.3;4.3)	0.092	1.5 (−0.8;3.8)	0.189
Heart rate (10.4 bpm)	**2.7 (0.3;5.1)**	**0.024**	0.7 (−1.6;3.2)	0.518	−0.3 (−2.7;2.1)	0.806
Total cholesterol (0.95 nmol/L)	−0.2 (−2.6;2.1)	0.823	−0.4 (−2.8;2.0)	0.735	−0.4 (−2.7;1.9)	0.702
LDL (0.83 nmol/L)	−0.7 (−3.1;1.7)	0.554	−0.5 (−3.0;1.8)	0.621	−0.7 (−3.0;1.6)	0.556
HDL (0.35 nmol/L)	**−2.5 (−5.0;−0.0)**	**0.046**	−2.0 (−4.6;0.4)	0.099	−1.1 (−3.6;1.3)	0.363
LDL/HDL cholesterol ratio (0.70)	1.2 (−1.2;3.7)	0.297	0.8 (−1.6;3.3)	0.498	0.1 (−2.8;2.4)	0.974
Triglycerides (0.79 nmol/L)	**3.7 (1.4;6.1)**	**0.002**	**3.2 (0.2;6.3)**	**0.036**	2.1 (−0.8;5.2)	0.148
Smoking (y/n)	**17.0 (11.0;23.1)**	**<0.001**	**14.0 (7.4;20.6)**	**<0.001**	**13.8 (7.5;20.2)**	**<0.001**
Alcohol intake (3 ordinal cat.)	**7.2 (3.4;10.9)**	**<0.001**	**6.3 (2.2;10.6)**	**0.003**	**6.0 (2.0;10.0)**	**0.004**
Physical exercise (3 ordinal cat.)	**−6.4 (−11.3;−1.5)**	**0.010**	−3.1 (−8.2;1.8)	0.204	−1.9 (−6.8;2.9)	0.423
Fruit intake (3 ordinal cat.)	**−4.1 (−8.4;0.0)**	**0.050**	−2.4 (−6.7;1.8)	0.259	−2.4 (−6.6;1.7)	0.235
CRP (quartiles)	**4.3 (2.3;6.4)**	**<0.001**	**4.7 (2.7;6.7)**	**<0.001**	**4.7 (2.7;6.7)**	**<0.001**

a) Models adjusted one by one for age and sex. b) Adjusted one by one for age, sex, smoking and alcohol intake. c) As b plus adjusted for CRP. Beta coefficients are expressed as increase of MMP-9 in ng/mL per SD, dichotomy or category, respectively.

Coefficients written in **bold** if significant. a) n = 335 to 345, b) and c) n = 313, based on participants with full info on all variables.

The partial correlation between MMP-9 and CRP (adjusting for age and sex) was r = 0.17 (n = 335) p<0.001. After exclusion for acute ongoing inflammation (CRP>10 mg/L) the coefficient was still fairly low, r = 0.24 (n = 329) p<0.001.

## Discussion

The main finding in this study is that there was a highly significant correlation between plasma levels of MMP-9 and total load of CV risk factors, in a population-based sample without reported symptomatic CAD. The association found was not driven by one single risk factor alone, and the trend remained significant after additional adjustment for CRP and TIMP-1. Furthermore, significant associations of MMP-9 levels were found with the risk factors SBP, smoking, alcohol intake and CRP in a multivariate regression.

A noteworthy difference from earlier studies was that plasma levels of MMP-9 were detectable in all participants. This could possibly be due to a relatively short period of time passing between sample date and analysis, as MMP-9 has been shown to degrade even at low temperature [31]. Although the ELISA technique were slightly different, a comparison of our results with the Framingham study [16] is appropriate as the population characteristics according to traditional risk factors (as shown in Table 1) were in the same range in both studies. As MMP-9 could be detected in only 20% of the participants in the Framingham study [16] (using a 2-site sandwich ELISA assay with a detection level of 4 ng/mL), it was concluded that MMP-9 was unlikely to be an informative biomarker in a low-risk population. Our findings, based on 100% detection, may indicate an altered conclusion. The data thus supports the idea that elevated levels plasma levels of MMP-9 before onset of disease may reflect an ongoing remodelling and early pathogenesis of the arterial wall and/or a low-grade systemic inflammation.

There is a current discussion on the use of novel biomarkers for cardiovascular risk stratification. In that light, the low but significant correlation between CRP and MMP-9 is of particular interest in our findings. This implies that CRP and MMP-9, at least to some extent, could be markers of different physiological pathways. This assumption was strengthened by the finding that total risk load and single risk factors remain significant when adjusting for CRP in the regression models, which implies that MMP-9 as a biomarker gives partly other information apart from redundant information of CRP measurements. Plausible pathways of potential interest for MMP-9 regulation such as antioxidant/oxidant imbalance [32], [33] and/or influences of different hormones (stress [34] and sex [35], [36]) should be further investigated.

Given the participation rate and the exclusion criteria constituted of self-reported diagnoses and use of hypolipidaemic drugs, the population tested is supposedly driven by a selection towards a healthy population. Such a selection would not distort the main findings in this study, as the selection if having any impact, would weaken the associations tested. It should be noted however that the interpretation relies on self-reported data. The agreement between self-reported diagnosis and medical record of myocardial infarction has been demonstrated to be high, both in terms of specificity and sensitivity [37], but as the participants were not examined invasively in this study, the presence of significant CAD cannot be excluded.

The major limitation with this study is that, whereas the association to total risk load was stable regardless of model used, the power may not be sufficient enough to fully evaluate the associations to single risk factors. While we found a number of associations shown in table 2, the inter-relation between the coefficients are hard to fully interpret, due to a limited number of participants. This is in particular the case when including several risk factors into the multivariate analyses. It should for instance be noted that smoking and alcohol intake, the behavioral factors that remained significant in a multivariate analysis, were highly clustered to other risk factors. Only a handful of participants (n = 5) had smoking or alcohol intake as the only present behavioral risk factor. In most of the participants, these factors appeared in combination with at least one of the other tested risk factors. Hence, it cannot be excluded that an effect from e.g. low physical activity and/or low fruit and vegetable intake as found in the crude analysis would be hidden in a multivariate analysis due to co-variation to alcohol and smoking.

Further, the only variables that did not reach significance or close to significance when adjusting for age and sex were total cholesterol, LDL, LDL/HDL ratio and diabetes. On one hand, it has been suggested before that MMP-9 does not reflect the lipid profile [38]. On the other hand the variation in the lipid profile variables is likely to be truncated by the exclusion criteria and the selection towards a healthy population. Diabetes relied on self-reported data, why interpretation should be done with cautiousness.

Another limitation could be that data on genetic polymorphisms is not included in the data set. Genetic polymorphisms could at least partially explain the variance found in the distribution of MMP-9 at individual level [13], and should be considered in further analyses.

Some authors have suggested that including/adjusting for TIMP-1 would enhance the physiologic information of MMP-9 [22], [39], [40]. Thus we tested this in a randomly selected sub-sample (n = 201). However, in this sub-sample the addition of TIMP did not change the associations between MMP-9 and total risk load, thus we did not perform TIMP-analyses in the remaining plasma samples.

In conclusion we found that in a population without reported symptomatic CAD, MMP-9 levels were associated with total CV risk load as well as with single risk factors. This was found also after adjustment for CRP. The participants will be followed prospectively for CAD events and thus it will be possible to investigate the importance of MMP-9 as a marker for future cardiovascular risk.

## References

[1] Libby P (2002). Inflammation in atherosclerosis.. Nature.

[2] Hansson GK (2005). Inflammation, atherosclerosis, and coronary artery disease.. N Engl J Med.

[3] Berk BC, Weintraub WS, Alexander RW (1990). Elevation of C-reactive protein in “active” coronary artery disease.. Am J Cardiol.

[4] Ridker PM, Hennekens CH, Buring JE, Rifai N (2000). C-reactive protein and other markers of inflammation in the prediction of cardiovascular disease in women.. N Engl J Med.

[5] Ridker PM, Glynn RJ, Hennekens CH (1998). C-reactive protein adds to the predictive value of total and HDL cholesterol in determining risk of first myocardial infarction.. Circulation.

[6] Haverkate F, Thompson SG, Duckert F (1995). Haemostasis factors in angina pectoris; relation to gender, age and acute-phase reaction. Results of the ECAT Angina Pectoris Study Group.. Thromb Haemost.

[7] Kovanen PT, Kaartinen M, Paavonen T (1995). Infiltrates of activated mast cells at the site of coronary atheromatous erosion or rupture in myocardial infarction.. Circulation.

[8] Saren P, Welgus HG, Kovanen PT (1996). TNF-alpha and IL-1beta selectively induce expression of 92-kDa gelatinase by human macrophages.. J Immunol.

[9] Galis ZS, Sukhova GK, Lark MW, Libby P (1994). Increased expression of matrix metalloproteinases and matrix degrading activity in vulnerable regions of human atherosclerotic plaques.. J Clin Invest.

[10] Loftus IM, Naylor AR, Goodall S, Crowther M, Jones L (2000). Increased matrix metalloproteinase-9 activity in unstable carotid plaques. A potential role in acute plaque disruption.. Stroke.

[11] Nikkari ST, Hoyhtya M, Isola J, Nikkari T (1996). Macrophages contain 92-kd gelatinase (MMP-9) at the site of degenerated internal elastic lamina in temporal arteritis.. Am J Pathol.

[12] Brown DL, Hibbs MS, Kearney M, Loushin C, Isner JM (1995). Identification of 92-kD gelatinase in human coronary atherosclerotic lesions. Association of active enzyme synthesis with unstable angina.. Circulation.

[13] Blankenberg S, Rupprecht HJ, Poirier O, Bickel C, Smieja M (2003). Plasma concentrations and genetic variation of matrix metalloproteinase 9 and prognosis of patients with cardiovascular disease.. Circulation.

[14] Newby AC (2006). Do metalloproteinases destabilize vulnerable atherosclerotic plaques?. Curr Opin Lipidol.

[15] Tayebjee MH, Lip GY, MacFadyen RJ (2005). Matrix metalloproteinases in coronary artery disease: clinical and therapeutic implications and pathological significance.. Curr Med Chem.

[16] Kameda K, Matsunaga T, Abe N, Fujiwara T, Hanada H (2006). Increased pericardial fluid level of matrix metalloproteinase-9 activity in patients with acute myocardial infarction: possible role in the development of cardiac rupture.. Circ J.

[17] Wagner DR, Delagardelle C, Ernens I, Rouy D, Vaillant M (2006). Matrix metalloproteinase-9 is a marker of heart failure after acute myocardial infarction.. J Card Fail.

[18] Derosa G, D'Angelo A, Scalise F, Avanzini MA, Tinelli C (2007). Comparison between metalloproteinases-2 and -9 in healthy subjects, diabetics, and subjects with acute coronary syndrome.. Heart Vessels.

[19] Wright JL, Tai H, Wang R, Wang X, Churg A (2007). Cigarette smoke upregulates pulmonary vascular matrix metalloproteinases via TNF-alpha signaling.. Am J Physiol Lung Cell Mol Physiol.

[20] Sillanaukee P, Kalela A, Seppa K, Hoyhtya M, Nikkari ST (2002). Matrix metalloproteinase-9 is elevated in serum of alcohol abusers.. Eur J Clin Invest.

[21] Yasmin, McEniery CM, Wallace S, Dakham Z, Pulsalkar P (2005). Matrix metalloproteinase-9 (MMP-9), MMP-2, and serum elastase activity are associated with systolic hypertension and arterial stiffness.. Arterioscler Thromb Vasc Biol.

[22] Tayebjee MH, Nadar S, Blann AD, Gareth Beevers D, MacFadyen RJ (2004). Matrix metalloproteinase-9 and tissue inhibitor of metalloproteinase-1 in hypertension and their relationship to cardiovascular risk and treatment: a substudy of the Anglo-Scandinavian Cardiac Outcomes Trial (ASCOT).. Am J Hypertens.

[23] Sundstrom J, Evans JC, Benjamin EJ, Levy D, Larson MG (2004). Relations of plasma matrix metalloproteinase-9 to clinical cardiovascular risk factors and echocardiographic left ventricular measures: the Framingham Heart Study.. Circulation.

[24] Hollman G, Kristenson M (2007). The prevalence of the metabolic syndrome and its risk factors in a middle-aged Swedish population - Mainly a function of overweight?. Eur J Cardiovasc Nurs.

[25] Friedewald WT, Levy RI, Fredrickson DS (1972). Estimation of the concentration of low-density lipoprotein cholesterol in plasma, without use of the preparative ultracentrifuge.. Clin Chem.

[26] Gerlach RF, Demacq C, Jung K, Tanus-Santos JE (2007). Rapid separation of serum does not avoid artificially higher matrix metalloproteinase (MMP)-9 levels in serum versus plasma.. Clin Biochem.

[27] Khani BR, Ye W, Terry P, Wolk A (2004). Reproducibility and validity of major dietary patterns among Swedish women assessed with a food-frequency questionnaire.. J Nutr.

[28] Kallings LV, Leijon M, Hellenius ML, Stahle A (2007). Physical activity on prescription in primary health care: a follow-up of physical activity level and quality of life.. Scand J Med Sci Sports.

[29] Turner NA, Aley PK, Hall KT, Warburton P, Galloway S (2007). Simvastatin inhibits TNFalpha-induced invasion of human cardiac myofibroblasts via both MMP-9-dependent and -independent mechanisms.. J Mol Cell Cardiol.

[30] Evans J, Powell JT, Schwalbe E, Loftus IM, Thompson MM (2007). Simvastatin attenuates the activity of matrix metalloprotease-9 in aneurysmal aortic tissue.. Eur J Vasc Endovasc Surg.

[31] Rouy D, Ernens I, Jeanty C, Wagner DR (2005). Plasma storage at −80 degrees C does not protect matrix metalloproteinase-9 from degradation.. Anal Biochem.

[32] Siwik DA, Pagano PJ, Colucci WS (2001). Oxidative stress regulates collagen synthesis and matrix metalloproteinase activity in cardiac fibroblasts.. Am J Physiol Cell Physiol.

[33] Kameda K, Matsunaga T, Abe N, Hanada H, Ishizaka H (2003). Correlation of oxidative stress with activity of matrix metalloproteinase in patients with coronary artery disease. Possible role for left ventricular remodelling.. Eur Heart J.

[34] Aljada A, Ghanim H, Mohanty P, Hofmeyer D, Tripathy D (2001). Hydrocortisone suppresses intranuclear activator-protein-1 (AP-1) binding activity in mononuclear cells and plasma matrix metalloproteinase 2 and 9 (MMP-2 and MMP-9).. J Clin Endocrinol Metab.

[35] Potier M, Karl M, Elliot SJ, Striker GE, Striker LJ (2003). Response to sex hormones differs in atherosclerosis-susceptible and -resistant mice.. Am J Physiol Endocrinol Metab.

[36] Potier M, Elliot SJ, Tack I, Lenz O, Striker GE (2001). Expression and regulation of estrogen receptors in mesangial cells: influence on matrix metalloproteinase-9.. J Am Soc Nephrol.

[37] Okura Y, Urban LH, Mahoney DW, Jacobsen SJ, Rodeheffer RJ (2004). Agreement between self-report questionnaires and medical record data was substantial for diabetes, hypertension, myocardial infarction and stroke but not for heart failure.. J Clin Epidemiol.

[38] Kalela A, Ponnio M, Koivu TA, Hoyhtya M, Huhtala H (2000). Association of serum sialic acid and MMP-9 with lipids and inflammatory markers.. Eur J Clin Invest.

[39] Orbe J, Fernandez L, Rodriguez JA, Rabago G, Belzunce M (2003). Different expression of MMPs/TIMP-1 in human atherosclerotic lesions. Relation to plaque features and vascular bed.. Atherosclerosis.

[40] Holven KB, Halvorsen B, Bjerkeli V, Damas JK, Retterstol K (2006). Impaired inhibitory effect of interleukin-10 on the balance between matrix metalloproteinase-9 and its inhibitor in mononuclear cells from hyperhomocysteinemic subjects.. Stroke.

